# Root traits and their potential links to plant ideotypes to improve drought resistance in common bean

**DOI:** 10.1007/s40626-017-0090-1

**Published:** 2017-08-31

**Authors:** Jose Polania, Charlotte Poschenrieder, Idupulapati Rao, Stephen Beebe

**Affiliations:** 1 Centro Internacional de Agricultura Tropical (CIAT), A.A. 6713, Cali, Colombia; 2 Lab Fisiología Vegetal, Facultad de Biociencias, Universidad Autónoma de Barcelona, Bellaterra, Spain; 3 Present address: Plant Polymer Research Unit, National Center for Agricultural Utilization Research, Agricultural Research Service, United States Department of Agriculture, 1815 North University Street, Peoria, IL 61604, USA

**Keywords:** Fine roots, N fixation ability, Root biomass, Root length, Water saver, Water spender

## Abstract

Drought stress limits growth and yield of crops, particularly under smallholder production systems with minimal use of inputs and edaphic limitations such as nitrogen (N) deficiency. The development of genotypes adapted to these conditions through genetic improvement is an important strategy to address this limitation. The identification of morpho-physiological traits associated with drought resistance contributes to increasing the efficiency of breeding programs. A set of 36 bean genotypes belonging to the Middle American gene pool was evaluated. A greenhouse study using soil cylinders was conducted to determine root vigor traits (total root length and fine root production) under drought stress. Two field trials were conducted to determinate grain yield, symbiotic nitrogen fixation (SNF) ability and other shoot traits under drought stress. Field data on grain yield and other shoot traits measured under drought were related with the greenhouse data on root traits under drought conditions to test the relationships between shoot traits and root traits. Response of root vigor to drought stress appeared to be related with ideotypes of water use (water savers and water spenders). The water spender ideotypes presented deeper root system, while the water saver ideotypes showed a relatively shallower root system. Increase in SNF ability under drought stress was associated with greater values of mean root diameter while greater acquisition of N from soil was associated with finer root system. We identified seven common bean lines (SEA 15, NCB 280, SCR 16, SMC 141, BFS 29, BFS 67 and SER 119) that showed greater root vigor under drought stress in the greenhouse and higher values of grain yield under drought stress in the field. These lines could serve as parents for improving drought resistance in common bean.

## 1 Introduction

Drought stress is one of the major abiotic constraints limiting agricultural productivity, particularly for smallholder systems. Drought affects different plant processes resulting in reduced gas exchange, crop growth and productivity (Araújo et al. 2015). At root system level, their response to drought stress could differ not only between species but also within species (Lynch 2013). Drought stress also impacts the pattern of water uptake and use depending on the origin and evolution of the species and the agro-climatic conditions faced by them (Blum 2015).

Drought is a major abiotic stress limitation for common bean (*Phaseolus vulgaris*) production, affecting around 60% of bean producing regions and generating losses in production from 10 to 100% (Polania et al. 2016c). It is expected that the world demand for legumes will increase in the future, not only in developing countries, but also in developed nations given the increasing trend towards healthy diets (Daryanto et al. 2015). In order to respond to the increase in demand, common bean has to face challenges that include higher temperatures and the associated increase in evapotranspiration combined with erratic and lower rainfall (Beebe et al. 2013). Different climate models predict that many drought stressed areas in Eastern and Southern Africa will become drier over the next decades (Jones and Thornton 2003; Williams et al. 2007; Rippke et al. 2016), exacerbating the limitations of bean production due to severe drought stress.

Different strategies must be developed to face these new challenges. A key approach is breeding of bean varieties resistant to drought to ensure food security in marginal areas. Defining the root and shoot level physiological mechanisms in response to drought helps to identify desirable traits and procedures for phenotyping populations and accelerating plant breeding for better yield under water shortage (Oos-terom et al. 2016). Several shoot and root traits improve resistance to drought (Araújo et al. 2015). However their contribution to superior grain yield depends on the type of drought (early, intermittent and terminal) and the agro-ecological conditions where the crop is grown (Rao et al. 2017). According to agro-ecological zones and types of drought, breeding should target different plant ideotypes such as, the isohydric (‘water saving’) plant model and the anisohydric (‘water spending’) plant model (Polania et al. 2016a). The water saving model might have an advantage in the harshest environments, whereas the water spending model will perform relatively better under more moderate drought conditions (Blum 2015). Effective use of water (EUW), as proposed by (Blum 2009), implies not only maximal soil moisture capture for transpiration but also decreased non-stomatal transpiration and minimal water loss by soil evaporation. In the water spending model, the EUW would be the main component to consider in plant breeding programs for improving adaptation to drought where there is potential for deep-rooted genotypes to access water deep in the soil profile that normally plants with superficial roots cannot access (Araus et al. 2002).

Roots play a vital role in the absorption of water and nutrients by plants. However the phenotypic evaluation of root traits under field conditions is labor intensive and expensive. Keeping this in mind, some rapid and cost effective methodology has been used to carry out root phenotypic evaluations, such as small soil cylinders under greenhouse conditions, which allow to evaluate several root traits under different types of abiotic stress (Polania et al. 2009; Butare et al. 2011, 2012). Phenotypic evaluations of root traits in common bean under drought stress have shown the importance of different rooting patterns, including deep rooting which allows access to water from deeper soil layers (Sponchiado et al. 1989; White and Castillo 1992; Lynch and Ho 2005; Polania et al. 2009, 2012; Beebe et al. 2013, 2014; Rao 2014; Burridge et al. 2016). Different ideotypes of root system have been proposed for better crop adaptation to individual and combined abiotic stress conditions (Yang et al. 2013; Rao et al. 2016). One of the root ideotypes proposed to optimize water and N acquisition is the “steep, cheap and deep - SCD” (Lynch 2013). One premise of this ideotype is that, the availability of water and nitrogen (N) is better in deeper soil strata over the growing season (Lynch 2013). This SCD ideotype includes: early root vigor, large root biomass, larger root surface area, greater N uptake capacity of root cells, greater water uptake through enhanced transpiration and greater association with organisms fixing N (Lynch 2013; Rao et al. 2016). However, it is noteworthy that some of the key root traits contributing to improved adaptation to soils with low fertility are increased fine root formation and root hairs (Eissenstat 1992; Lynch 2011, 2013; Rao et al. 2016 Fine roots and root hairs can explore a large volume of soil and have a low carbon and energy requirement for their function (Eissenstat 1992; Huang and Fry 1998; Polania et al. 2009; Butare et al. 2011; Lynch 2011, 2013; Rao et al. 2016).

Increased capacity for water and nutrient uptake and higher crop growth rate must be accompanied by an improved harvest index (HI). Better remobilization of photosynthates to the grains is essential for the success of high yielding genotypes under drought stress (Polania et al. 2016c; Rao et al. 2017). Several studies using common bean have demonstrated the importance of better plant growth, accompanied by a superior photosynthate remobilization from plant structures to pod formation (pod partitioning index), and subsequently to grain filling and yield (pod harvest index) in improving adaptation to drought and also adaptation to low fertility soils (Beebe et al. 2013; Rao et al. 2013; Assefa et al. 2013; Yang et al. 2013; Beebe et al. 2014; Araújo et al. 2015; Polania et al. 2016c). Moreover it is possible to improve symbiotic nitrogen fixation (SNF) ability of common bean during drought stress, through the identification of genotypes that present greater ability to fix nitrogen under stress conditions (Devi et al. 2013; Polania et al. 2016b).

Strategic combination of different shoot and root traits seems to be the key in further improving adaptation to drought in common bean (Araújo et al. 2015). For this reason it is important to identify the role of root traits in improving adaptation to drought using the same group of bean genotypes and to test the relationships between root traits and shoot traits. Moreover, testing the relationships between root traits, SNF ability, and nutrient acquisition under drought stress may further contribute to improve sustainable bean production under water shortage.

The main objectives of this study were to: (i) determine genotypic differences in root vigor under drought stress and test the relationships between root vigor and grain yield and SNF ability; and (ii) identify a few promising genotypes that combine root vigor with greater values of SNF ability and grain yield under drought stress which could serve as parents in breeding programs aimed to improve drought resistance in common bean.

## 2 Materials and methods

### 2.1 Plant material

For this study 36 bush bean genotypes belonging to the Middle American gene pool were selected: twenty-two elite lines of common bean (BFS 10, BFS 29, BFS 32, BFS 67, MIB 778, NCB 226, NCB 280, RCB 273, RCB 593, SCR 16, SCR 2, SCR 9, SEN 56, SER 118, SER 119, SER 125, SER 16, SER 48, SER 78, SMC 141, SMC 43 and SXB 412); five interspecific lines between elite line SER 16 and *Phaseolus coccineus* (ALB 6, ALB 60, ALB 74, ALB 88 and ALB 213); one landrace of tepary bean (*Phaseolus acutifolius*) G 40001 from Veracruz-Mexico, and two interspecific lines between tepary bean and common bean (INB 841 and INB 827) developed from five cycles of congruity backcrossing of tepary with ICA Pijao (Mejía-Jiménez et al. 1994). SEA 15 and BAT 477 were included as drought resistant checks, and three commercial cultivars of common bean (DOR 390, Pérola and Tio Canela) as drought sensitive materials. BAT 477 NN was included as a non-nodulating bean genotype.

BFS (small red) lines have been developed to improve adaptation to low soil fertility and drought. SER, SCR and RCB (small red), SEN (small black) and NCB (small black) lines have been developed for improved adaptation to drought, disease resistance and commercial grain. ALB (small red) lines were developed for improved adaptation to drought and aluminum toxicity in acidic soil.

### 2.2 Root phenotyping using soil cylinder system

A greenhouse study was conducted at the main experiment station of the International Center for Tropical Agriculture (CIAT) in Palmira, Colombia, using transparent plastic cylinders (120 cm long, 7.5 cm diameter) filled with a Mollisol from Palmira, Colombia (Polania et al. 2009; Butare et al. 2011). Soil cylinders were carefully packed with soil: sand mixture (2:1), with a final bulk density of 1.4 g cm^–3^. The seeds were germinated in paper towels and uniform seedlings were selected for transplanting to transparent plastic cylinders, each of which was inserted into PVC sleeve-tubes. Plants were grown for 45 days in these plastic cylinders/PVC sleeve-tubes with an average maximum and minimum temperature of 34 and 21 °C. A randomized complete block design (RCB) with three replications was used. One level of water supply treatment was applied: progressive soil drying with no watering after 10 days of growth in order to simulate terminal drought stress conditions. The initial soil moisture was at 80% of field capacity. Plants received no water application and each cylinder was weighed at 2 day intervals to determine the decrease in soil moisture content until the time of plant harvest.

Plants were harvested at 45 days after transplanting (35 days of withholding of water application to induce water-stress treatment) when the plants are at early pod development. The root traits were evaluated at this time since the greatest phenotypic differences between the genotypes were observed (Polania et al. 2009; Butare et al. 2012). Visual rooting depth (VRD) was measured during the experiment at 7 day intervals using a ruler with cm scale, registering the total depth reached by the roots that were visible through the plastic cylinder. Root growth rate per day was calculated. At harvest, leaf area (LICOR model LI-3000), shoot biomass and root production were measured. The roots in each cylinder were washed free of soil and sand. The washed roots were scanned as images by a desk scanner (Epson expression 1680 professional). From the scanned images, total root length (m plant^–1^) and proportion of fine roots or proportion of roots (%) with diameter less than 0.5 mm, were estimated through image analysis using WinRHIZO software (Regent Instruments Inc., Quebec, Canada). Total root and shoot dry weight per plant were determined after the roots and shoots were dried in an oven at 60 °C for 48 h.

### 2.3 Shoot phenotyping under field conditions

Complete data on shoot phenotyping from field trials were reported previously (Polania et al. 2016a, b). Two field trials were conducted during the dry season (from June to September in both 2012 and 2013), at CIAT in Palmira, Colombia. The soil is a Mollisol (Aquic Hapludoll) with adequate nutrient supply. During the crop-growing season, maximum and minimum air temperatures in 2012 were 31.0 and 19.0 °C, and in 2013 were 30.2 and 19.2 °C, respectively. Total rainfall during the active crop growth was 85.8 mm in 2012 and 87.7 mm in 2013. The potential pan evaporation was of 385.2 mm in 2012 and 351.0 mm in 2013. Two levels of water supply (irrigated and rainfed) were applied to simulate well-watered (control) and drought stress treatments respectively. Trials were furrow irrigated (approximately 35 mm of water per irrigation). The drought stress treatment under rainfed conditions in 2012 received 3 irrigations (at 3 days before planting and at 5 and 23 days after planting) and in 2013 also received 3 irrigations (at 3 days before planting and at 4 and 15 days after planting). In both years, irrigation was suspended after the third irrigation to induce terminal drought stress conditions. The irrigated control treatment received 5 irrigations in 2012 and 6 irrigations in 2013 to ensure adequate soil moisture for crop growth and development. Experimental units consisted of 4 rows with 3.72 m row length with a row-to-row distance of 0.6 m and plant-to-plant spacing of 7 cm (Polania et al. 2016a).

Grain yield and other shoot traits such as: days to flowering (DF), days to physiological maturity (DPM), canopy biomass (CB), leaf area index (LAI), stomatal conductance (SC), seed number per area (SNA), pod number per area (PNA), harvest index (HI), pod harvest index (PHI), pod partitioning index (PPI), grain carbon isotope discrimination (CID-G), percentage of N derived from the atmosphere using grain tissue (%Ndfa-G), percentage of N derived from soil using grain tissue (%Ndfs-G) and total shoot and seed N content per unit area (kg ha^–1^) were recorded for each plot. Leaf area was measured using a leaf area meter (model LI-3000, LI-COR, NE, USA) and the leaf area index (LAI) was calculated as leaf area per unit land area. The stomatal conductance was measured with a portable leaf porometer (Decagon SC-1) on a fully expanded young leaf of one plant within each replication. The following attributes were determined according to Beebe et al. (2013): harvest index (HI) (%): seed biomass dry weight at harvest/total shoot biomass dry weight at mid-pod filling × 100; pod harvest index (PHI) (%): seed biomass dry weight at harvest/pod biomass dry weight at harvest × 100; pod partitioning index (PPI) (%): pod biomass dry weight at harvest/total canopy biomass dry weight at mid-pod filling × 100 (Polania et al. 2016a, b).

### 2.4 Statistical analysis

All data were analyzed using the SAS (v 9.0) PROC MIXED and PROC CORR (SAS Institute Inc. 2008). The adjusted means for each genotype in each trial (field and greenhouse) were obtained using the mixed models theory together with the MIXED procedure (SAS Institute Inc. 2008) considering the effects of the replications and blocks within replications as random and genotypes as fixed. Pearson correlation coefficients between grain yield and other shoot traits that had been determined under field conditions with root traits measured under greenhouse conditions using a soil cylinder system were calculated by the PROC CORR (SAS Institute Inc. 2008) using mean values per genotype per trial (field and greenhouse). Principal component analysis (PCA) with data on root traits (VRD43: visual rooting depth at day 43 after planting, RGR: root growth rate, TRB: total root biomass, TRL: total root length, MRD: mean root diameter, RV: root volume and FRP: fine root proportion) and shoot traits (DF: days to flowering, DPM: days to physiological maturity, SC: leaf stomatal conductance, CID-G: carbon isotope discrimination-grain, LAI: leaf area index, CB: canopy biomass, PHI: pod harvest index, PPI: pod partitioning index, HI: harvest index, GY: grain yield, 100SW: 100 seed weight, SNA: seed number per area, PNA: pod number per area, %Ndfa-G: % N derived from the atmosphere using grain, %Ndfs-G: % N derived from the soil using grain, N-Upt-SH: shoot N uptake, N-Upt-G: grain N uptake, TNdfa-G and grain N fixed) was performed based on the correlation matrix using the PRINCOMP (principal components) procedure from SAS (SAS Institute Inc. 2008). Simple scatter graph (x, y pair) between grain yield vs total root length, grain yield vs total root biomass, and total root length and fine root proportion were realized using SigmaPlot software.

## 3. Results

### 3.1 Genotypic differences in root vigor under drought stress

Based on genotypic differences in grain carbon isotope discrimination (CID-G), leaf stomatal conductance, canopy biomass, and grain yield under drought stress, the lines resistant to drought conditions were classified into two groups, water savers (G 40001, SER 16, ALB 60, ALB 6, BFS 10) and water spenders (NCB 280, NCB 226, SEN 56, SCR 16) (Polania et al. 2016a). Results obtained on root vigor under drought stress in the greenhouse are shown in Table 1. Significant differences (*P* < 0.05) between genotypes were observed in visual root growth rate under drought conditions. Water spender lines were superior in their visual root growth rate under drought conditions and these were considered as genotypes with high root vigor (Table 1). Water saver and drought susceptible lines showed lower root growth rate under drought stress and these were considered as genotypes with low root vigor (Table 1). These two groups of genotypes also presented the highest and lowest visual rooting depth, respectively, at 43 days after planting in the greenhouse and grown under drought stress.

**Table 1:** Phenotypic differences in visual rooting depth at 43 days after planting (VRD43), root growth rate per day (RGR), mean root diameter (MRD), % N derived from the atmosphere using grain tissue (%Ndfa-G), % N derived from the soil using grain tissue (%Ndfs-G) and shoot N uptake (N-Upt-SH) of bean genotypes classified such as water saver, moderate water saver, water spender and drought susceptible when grown under drought conditions at CIAT-Palmira, Colombia**

Genotype	VRD43 (cm plant^–1^)	RGR (mm day^–1^)	MRD (mm)	%Ndfa-G	%Ndfs-G	N-Upt-SH (kg ha^–1^)
Water savers						
G 40001	39	9	0.56	8	92	61
SER 16	42	10	0.63	18	82	66
ALB 6	49	11	0.74	22	78	48
BFS 10	38	9	0.65	18	82	61
Moderate water savers						
BFS 29	43	10	0.62	26	74	67
SEA 15	55	13	0.55	20	80	49
RCB 593	40	9	0.64	35	65	52
SER 125	45	10	0.70	29	71	61
Water spenders						
NCB 280	57	13	0.53	22	78	59
NCB 226	47	11	0.51	23	77	51
SEN 56	37	9	0.76	20	80	56
SCR 16	48	11	0.65	26	74	60
Drought sensitive						
MIB 778	37	9	0.50	12	88	38
Perola	47	11	0.61	22	78	35
DOR 390	51	12	0.55	18	82	47
Tio Canela 75	40	9	0.58	26	74	42
Mean	45	10	0.63	20	80	53
Sig. diff.	_*_	_*_	_*_	_*_	_*_	_*_

* Significant difference at 0.05 level as estimated from the MIXED procedure

** The genotypes were classified such as water saver, moderate water saver, water spender based on genotypic differences in grain carbon isotope discrimination (CID-G), leaf stomatal conductance, canopy biomass, and grain yield under drought stress (Polania et al. 2016a)

**Fig. 1:**
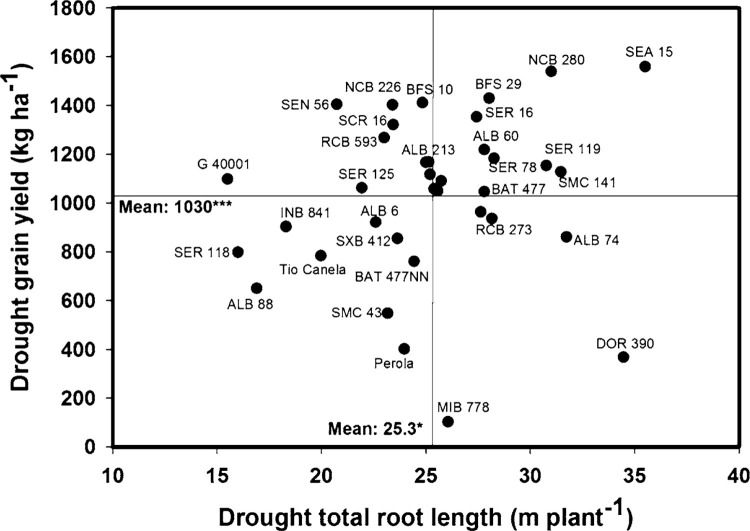
Identification of genotypes with greater values of grain yield (field conditions) and total root length (greenhouse conditions) under drought stress in Palmira. Higher yielding genotypes with greater values of total root length were identified in the *upper, right hand quadrant.* ***,* Significant difference at 0.001 and 0.05 probability level as estimated from the MIXED procedure

A wide range of diversity and significant genotypic differences (*P* < 0.05) in total root length were observed under drought conditions (Fig. 1). Eight lines (SEA 15, NCB 280, BFS 29, SER 16, SER 119, ALB 60, SMC 141, SER 78) combined higher values of total root length with superior grain yield under drought stress (Fig. 1). The lines ALB 88, Tio Canela 75, SMC 43 and Perola showed less root vigor with lower grain yield under drought stress (Fig. 1). In contrast to the above four genotypes, seven genotypes (G 40001, SEN 56, NCB 226, SCR 16, RCB 593, SER 125, BFS 10) were superior in their grain yield under drought stress with less root vigor compared with the other genotypes tested (Fig. 1). Contrastingly, two genotypes MIB 778 and DOR 390 showed greater root vigor but lower grain yields under drought stress (Fig. 1).

**Fig. 2:**
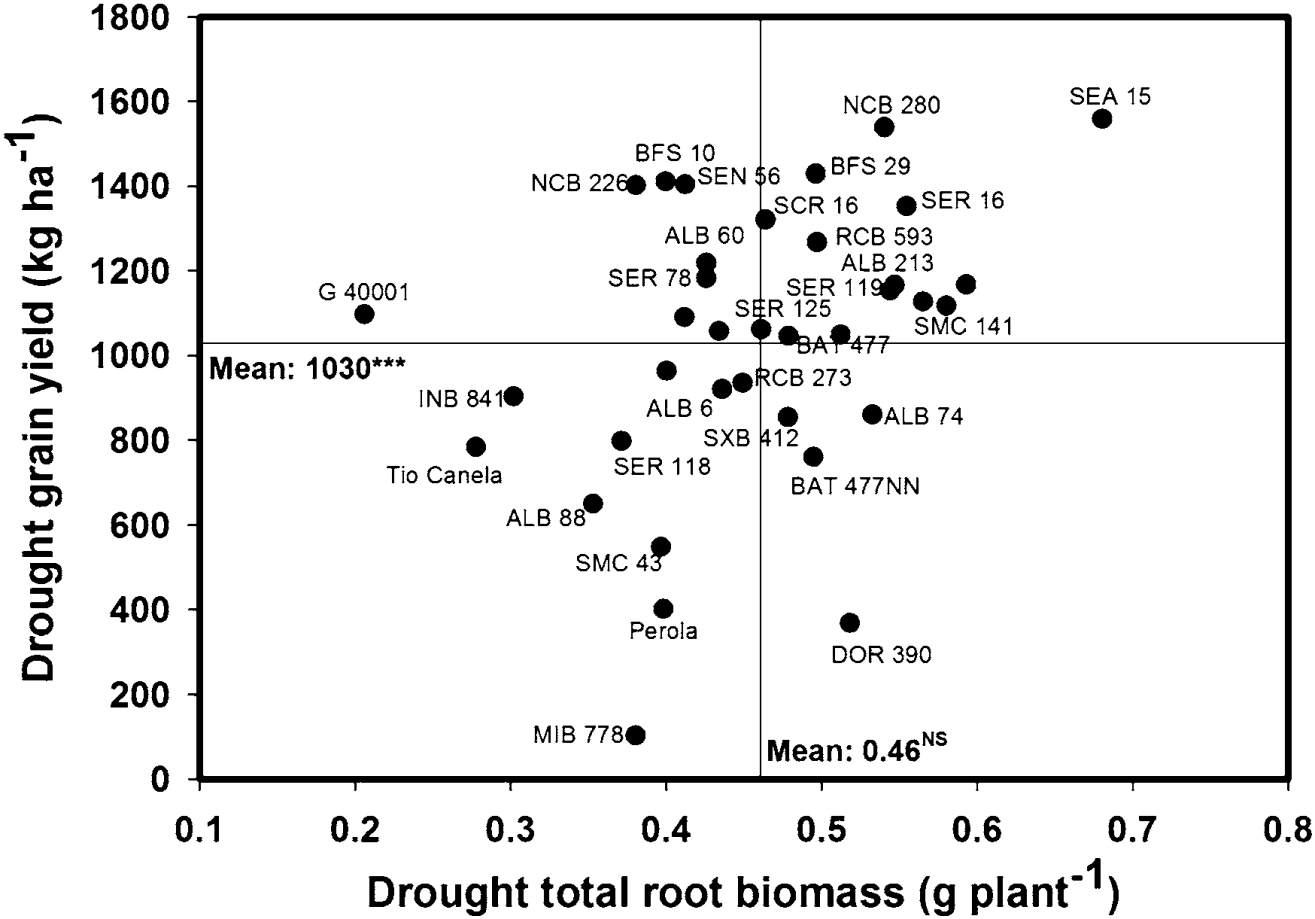
Identification of genotypes with greater values of grain yield (field conditions) and total root biomass (greenhouse conditions) under drought stress in Palmira. Higher yielding genotypes with greater values of root biomass were identified in the *upper, right hand quadrant*. *** Significant difference at 0.001 probability level as estimated from the MIXED procedure

Eight lines (SEA 15, NCB 280, BFS 29, SER 16, SER 119, RCB 593, ALB 213, SMC 141) combined higher values of total root biomass with superior grain yield under drought stress (Fig. 2). Five genotypes (ALB 88, Tio Canela 75, SMC 43, MIB 778, Perola) showed lower values of root biomass with lower values of grain yield under drought stress (Fig. 2). Four genotypes (G 40001, SEN 56, NCB 226, BFS 10) were superior in their grain yield under drought stress and these also showed lower values of root biomass compared to the other lines tested (Fig. 2). The commercial check DOR 390 presented greater root vigor in terms of root biomass but lower grain yield under drought stress (Fig. 2).

**Fig. 3:**
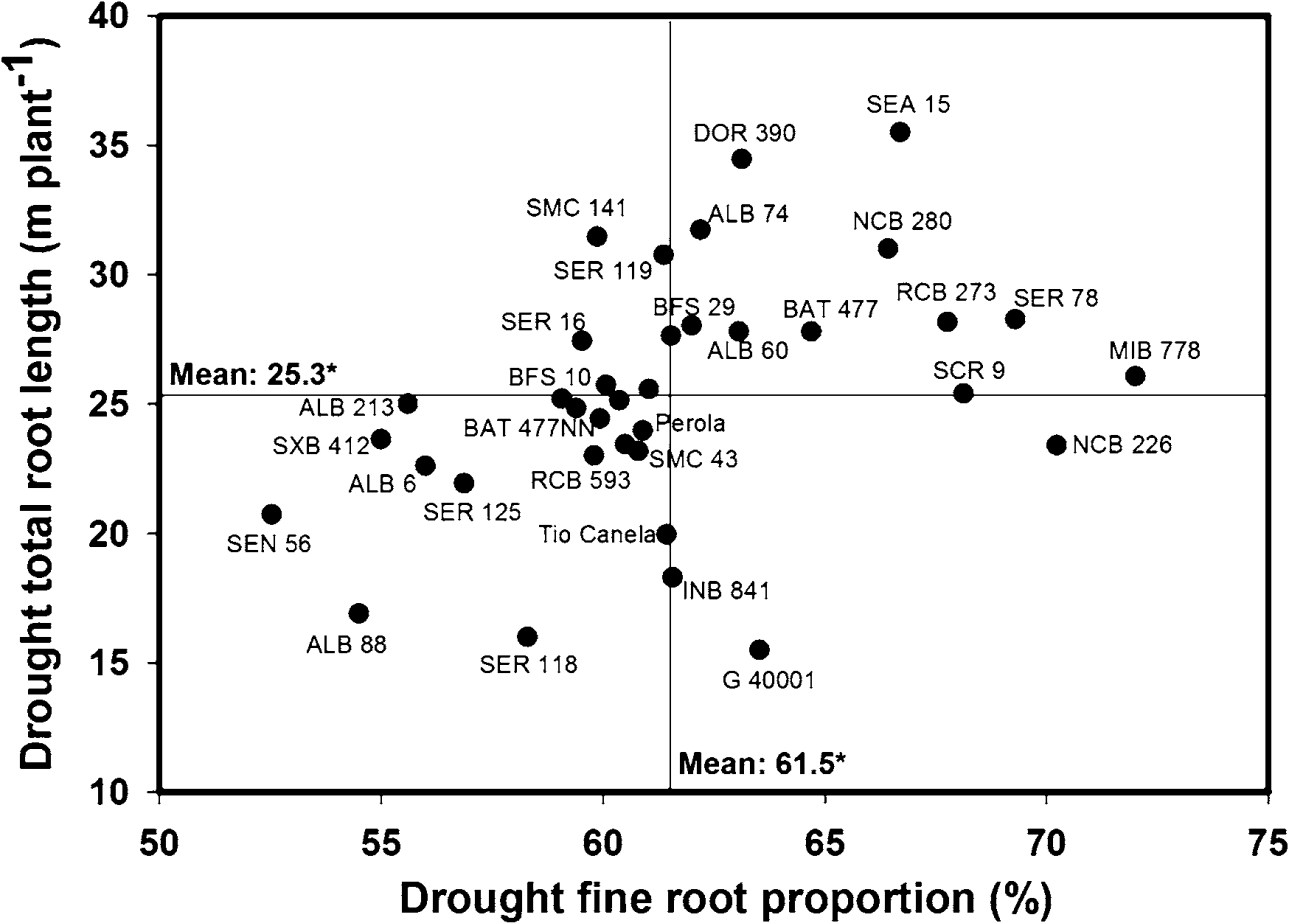
Identification of genotypes with greater values of total root length (TRL) and fine root proportion (FRP) under drought stress in Palmira. Higher TRL genotypes with greater values of FRP were identified in the *upper, right hand quadrant*. ***,* Significant difference at 0.001 and 0.05 probability level as estimated from the MIXED procedure

Significant genotypic differences (*P* < 0.05) were observed in fine root proportion under drought stress conditions. Ten genotypes (MIB 778, NCB 226, SER 78, SCR 9, RCB 273, SEA 15, NCB 280, BAT 477, G 40001, DOR 390) developed fine roots under drought stress (Fig. 3). Twelve lines (SMC 141, RCB 593, SER 16, BFS 10, BFS 67, SER 118, SER 125, ALB 6, ALB 213, SXB 412, ALB 88, SEN 56) developed a greater proportion of roots with greater average root diameter (Fig. 3). The accession of P. *acutifolius* (G 40001) and its inter-specific progeny INB 841 presented a fine root system but with relatively low values of total root length and total root biomass under drought stress (Figs. 1, 2, 3).

### 3.2 Relationship between root vigor and shoot traits including grain yield

A positive and significant correlation was observed between different root traits and grain yield under drought conditions (Table 2). Grain yield was correlated with total root biomass (*r* = 0.28**), total root length (*r* = 0.22*) and total root volume (*r* = 0.22*). Also a significant and positive correlation (*r* = 0.58***) was observed between total root length and fine root proportion (Table 2). A negative relationship was observed between CID-G and fine root proportion under drought conditions (*r* = -0.27**) (Table 2). No correlation was observed between fine root proportion and grain yield under drought stress. Several lines with superior total root length showed higher proportion of fine roots under drought conditions (Fig. 3).

**Table 2:** Correlation coefficients (r) among visual root growth rate in mm day^–1^ (RGR), total root biomass in g plant^–1^ (TRB), total root length in m plant^–1^ (TRL), mean root diameter in mm (MRD), total root volume in cm^3^ (TRV), fine root proportion in % (FRP) observed under drought stress in the greenhouse and canopy biomass in kg ha^–^ (CB), grain yield in kg ha^–1^ (GY) and grain C isotope discrimination in % (CID-G) observed under drought stress in the field of 36 bean genotypes grown at CIAT-Palmira, Colombia

	RGR	TRB	TRL	MRD	TRV	FRP	CB	GY	CID-G
RGR	1.00								
TRB	0.47***	1.00							
TRL	0.46***	0.72***	1.00						
MRD	–0.11	–0.07	–0.61***	1.00					
TRV	0.38***	0.68***	0.38***	0.39***	1.00				
FRP	0.07	0.09	0.58***	–0.95***	0.40***	1.00			
CB	0.00	0.29**	0.29**	–0.20*	0.09	0.20*	1.00		
GY	0.12	0.28**	0.22*	–0.05	0.22*	0.04	0 70***	1.00	
CID-G	–0.02	0.08	–0.12	0.29**	0.25*	–0.27**	0.32***	0.45***	
									1.00

Correlation coefficients were calculated by the PROC CORR. Results marked with *, **, *** are significant at the 0.05, 0.01 and 0.001 probability levels, respectively

Positive and significant correlations under drought stress conditions were observed between: mean root diameter and %Ndfa-G (*r* = 0.43***); fine root proportion and %Ndfs (*r* = 0.46***); fine root proportion and shoot N uptake in kg ha^–1^ (*r* = 0.37***); and total root length and shoot N uptake in kg ha^–1^ (*r* = 0.39***). Seven lines (NCB 226, SER 78, SCR 9, SEA 15, NCB 280, BAT 477, G 40001) combined fine root system development (Fig. 3) with superior N uptake from the soil under drought stress (Table 1). Nine lines (SMC 141, RCB 593, SER 16, BFS 10, BFS 67, SER 125, ALB 6, SXB 412, SEN 56) combined a greater value of average root diameter (Fig. 3) with better symbiotic nitrogen fixation (SNF) ability (Table 1) under drought stress conditions. Five lines (NCB 280, BFS 29, SER 16, SER 119, BAT 477) combined higher values of total root length (Fig. 1) with higher values of shoot N uptake (Table 1) under drought stress conditions.

Multivariate analysis showed that the first three components of PC analysis could explain 61% of the variability observed in the shoot and root phenotyping of 36 bean lines under drought conditions (Table 3, Fig. S1). In component 1, the traits with the largest contribution to variability were: grain yield, canopy biomass, pod harvest index, harvest index, seed number per area, total N uptake using grain tissue and total N fixed from atmosphere using grain tissue for estimation (Table 3). In component 2, the traits with the largest contribution to variability were: visual rooting depth at day 43 after planting, root growth rate, total root biomass, total root length and root volume (Table 3). The PC analysis showed that under drought conditions, yield was primarily associated with canopy biomass, pod harvest index, harvest index, seed number, N derived from the atmosphere and N derived from the soil. A negative association of yield under drought was associated with days to flowering (Table 3). Yield was also associated with root vigor such as visual rooting depth, root growth rate, total root length, total root biomass and root volume (Table 3). The PC analysis showed that grain yield under drought stress conditions is associated with earliness, root vigor, superior plant growth, increase in partitioning of dry matter to grain and seed number per area.

**Table 3:** Eigen values and percent of total variation and component matrix for the principal component axes

Principal components	1	2	3	4	5	6	7
Eigen values	9.67	3.88	3.05	2.33	2.30	1.39	1.09
% of variance	36	14	11	9	9	5	4
Cumulative	36	50	61	70	79	84	88
Component matrix							
DF	**–0.213**	0.143	0.176	0.014	0.137	**0.314**	0.237
DPM	–0.187	0.195	0.183	–0.018	0.159	**0.422**	0.163
SC	0.134	0.152	0.044	0.093	**0.295**	0.083	**–0.504**
CID-G	0.146	0.034	**0.265**	–0.080	0.210	0.226	–0.140
LAI	0.137	0.186	–0.012	0.226	–0.023	**0.429**	**0.396**
CB	**0.255**	0.008	–0.241	0.090	–0.186	0.122	0.085
PHI	**0.251**	–0.111	0.067	–0.023	0.142	–0.315	0.184
PPI	0.209	–0.094	**0.285**	–0.098	0.216	0.043	–0.087
HI	**0.266**	–0.104	0.158	–0.116	0.180	0.043	–0.060
GY	**0.303**	–0.012	–0.067	0.049	–0.032	–0.013	–0.151
100SW	0.124	0.238	0.238	–0.053	–0.204	0.212	**–0.376**
SNA	**0.257**	**–0.207**	–0.057	–0.068	0.161	0.033	0.173
PNA	0.247	–0.174	0.001	–0.010	0.192	0.150	0.208
%Ndfa-G	0.152	0.033	0.232	**–0.328**	**–0.361**	–0.020	0.164
%Ndfs-G	–0.152	–0.033	–0.232	**0.328**	**0.361**	0.020	–0.164
N-Upt-SH	0.221	–0.093	**–0.269**	0.181	–0.063	0.102	0.134
N-Upt-G	**0.279**	–0.005	–0.149	0.180	0.020	0.058	–0.129
TNdfa-G	**0.257**	0.054	0.097	–0.149	**–0.300**	0.077	–0.082
VRD43	0.093	**0.362**	–0.038	–0.124	**0.250**	**–0.252**	0.192
RGR	0.095	**0.361**	–0.036	–0.125	**0.251**	**–0.251**	0.190
TRB	0.126	**0.396**	–0.054	0.198	–0.066	0.000	–0.048
TRL	0.064	**0.408**	–0.187	–0.090	–0.037	–0.088	0.004
MRD	0.026	–0.074	**0.345**	0.469	**–0.103**	–0.124	0.047
RV	0.075	0.324	0.104	0.306	–0.171	–0.196	0.016
FRP	–0.039	0.105	**–0.338**	**–0.442**	0.087	0.158	–0.119

*DF* days to flowering, *DPM* days to physiological maturity, *SC* leaf stomatal conductance, *CID-G* carbon isotope discrimination-grain, *LAI* leaf area index, *CB* canopy biomass, *PH*I pod harvest index, *PPI* pod partitioning index, *HI* harvest index, *GY* grain yield, 100*SW* 100 seed weight, *SNA* seed number per area, *PNA* pod number per area, %*Ndfa-G* % *N* derived from the atmosphere using grain, %*Ndfs-G* % *N* derived from the soil using grain, *N*-*Upt-SH* shoot N uptake, *N*-*Upt-G* grain N uptake, *TNdfa-G* grain N fixed, *VRD43* visual rooting depth at day 43 after planting, *RGR* root growth rate, *TRB* total root biomass, *TRL* total root length, *MRD* mean root diameter, *RV* root volume, *FRP* fine root proportion

## 4 Discussion

### 4.1 Genotypic differences in root vigor and its relationship with grain yield

This study evaluated the role of a number of shoot and root traits in improving adaptation to drought in advanced bean lines developed over several cycles of breeding. The results from this study showed marked diversity in root system development under drought conditions. Bean lines with low grain yield under field conditions were characterized by poor root vigor, with a low rate of root growth and shallow root development under drought conditions. Genotypes with superior grain yield under drought stress under field conditions and classified as water spenders (Polania et al. 2016a) showed higher root vigor with deeper rooting ability under drought stress in the greenhouse. Deep roots may develop from the basal roots that change their root angle to turn downward, or from lateral roots that develop from a tap root, or both (Bonser et al. 1996; Ho et al. 2005; Basu et al. 2007; Lynch 2011; Miguel et al. 2013; Beebe et al. 2014; Burridge et al. 2016).

High root vigor and deeper rooting ability in water spender type genotypes allows the plant to access greater amounts of available water, permitting the processes of gas exchange to continue, with the accumulation of water soluble carbohydrates in the stem and their subsequent remobilization to grain filling as was observed in some wheat genotypes (Lopes and Reynolds 2010). When this ability to extract water is combined with a better photosynthate partitioning towards grain, this results in improved grain yield under drought stress. On the other hand, the commercial check DOR 390 with its high root vigor and deeper rooting ability appears to allocate greater proportion of carbon to root growth at the expense of grain production under drought stress. Results on DOR 390 showed that a vigorous root system without the adequate combination of other desirable plant attributes such as better plant growth and improved partitioning of dry matter to grain results in poor adaptation to drought stress. It is also notable that although the line SER 16 and its progeny ALB 60 were classified as water savers (Polania et al. 2016a), they showed a deeper and more vigorous root system under drought stress, suggesting that even when water can be accessed by deep rooting, stomatal regulation may still function as a key mechanism in a water saving strategy. The ability of SER 16 to regulate transpiration was reported in a previous study conducted under greenhouse conditions where this line was characterized as responsive to progressive soil drying under greenhouse conditions by closing its stomata sooner than the other genotypes (Devi et al. 2013).

The genotypes with superior grain yield under drought stress under field conditions and classified as water savers (Polania et al. 2016a) presented moderate root vigor with slower root growth resulting in a shallower root system under drought stress in the greenhouse (Table 2, Fig. 1). These genotypes showed a strategy of conserving water for higher WUE, combined with a better remobilization of photosynthates to grain formation, resulting in better performance under drought stress. The strategy of these water saving genotypes may be associated with shoot traits related with conserving water at the vegetative growth stage, such as lower leaf conductance, smaller leaf size, and lower leaf area index. These traits would make more water available for reproductive development and grain filling, resulting in better grain yield under terminal drought stress conditions (Zaman-Allah et al. 2011; Araújo et al. 2015).

Thus selection only based on root system characteristics is not enough without the proper combination of other desirable shoot traits. It is important to determine what size and what kind of distribution of root system across soil profile is required for a specific type of soil and specific type of drought to minimize trade-offs or any restriction to shoot growth and yield (Bingham 2001).

### 4.2 Relationship between root vigor and shoot traits including grain yield under drought stress

The results from this study showed some significant relationship between the type of root system and the ability of the plant for SNF and also to acquire mineral N from the soil under drought stress. Several genotypes showed the ability to combine superior grain production under drought stress with better SNF ability and increased presence of greater average root diameter. This relationship could be due to an increased carbon supply to nodules under drought stress from the stored carbohydrates in thicker roots. Large root diameter is known to correlate with greater sink strength (Thaler and Pages 1996). Previous evaluations with BAT 477, showed that this line maintained a relatively higher level of SNF under drought stress; possibly due to a deep and vigorous root system that accessed water from deeper soil layers to avoid drought and to alleviate stress on SNF process (Castellanos et al. 1996; Araújo et al. 2015). Moreover, the positive relationship observed between fine roots proportion and mineral N uptake from the soil, highlights the importance of fine root system to acquire mineral N from soil. The production of fine roots can be a strategy to facilitate absorption of water and mineral N when the available water in soil is limited; fine roots are “economical to build” and are essential for acquiring water and nutrients due to their high surface area per unit mass (Eissenstat 1992; Huang and Fry 1998).

A very vigorous root system contributes to greater acquisition of water and nutrients to support the vegetative growth of the shoot but if this is not combined with greater ability to partition dry matter to grain, this could lead to poor grain yield under drought stress. Thus, a vigorous and deeper root system, with rapid growth rate is useful but not enough to have resistance to drought in common bean. Our results indicate that for water spender type of genotypes, a strategic combination of root and shoot traits such as deep root system combined with the ability to remobilize photosynthates from vegetative structures to the pods and subsequently to grain production could contribute to superior performance under intermittent drought stress (Beebe et al. 2014; Rao 2014). It also appears that for water saving genotypes, with a combination of development of fine root system with high water use efficiency mechanisms at leaf level will contribute to improved adaptation to prolonged or terminal drought stress (Polania et al. 2016a).

In common bean, a universal ideotype of genotype with adaptation to drought would not be appropriate to target to diverse agroecological niches in the tropics. There is need to develop ideotypes of bean adapted to drought according to the type of drought, climate and soil. Phenotypic evaluation of shoot traits under field conditions (Polania et al. 2016a, b) and root traits under greenhouse conditions allowed the classification of the genotypes tested into two groups, water savers and water spenders, that allows for targeting to specific agro-ecological niches. This effort also contributes to the identification of morpho-physiological shoot and root traits that are associated with each group. The water spender genotypes should be useful for cultivation in areas exposed to intermittent drought stress in Central America, South America, and Africa, particularly in agro-ecological regions where rainfall is intermittent during the season and soils that can store greater amount of available water deep in the soil profile. The main morpho-physiological characteristics of the water spender and water saver type of genotypes are summarized in Table 4. The water saver genotypes can be more suitable for farmers in semiarid to dry environments dominated by terminal type of drought stress and soils with limited water available.

**Table 4:** Root and shoot traits related to the water saving ideotype and the water spending ideotype proposed for targeting improved common bean genotypes to drought prone agroecological zones

Ideotypes	Water savers’ ideotype	Water spenders’ ideotype
Root and shoot traits	Intermediate to shallow rooting system	Vigorous and deep rooting system
	Intermediate root growth rate and penetration ability	Rapid root growth rate and penetration ability
	Fine root system	Thicker root system
	Lower SNF ability	Moderate SNF ability
	Earliness	Earliness
	High water use efficiency	Effective use of water
	Reduced transpiration rate	Moderate transpiration rate
	Less carbon isotope discrimination	More carbon isotope discrimination
	Limited leaf area and canopy biomass development	Moderate canopy biomass accumulation
	Reduced sink strength	Moderate sink strength
Targeting to specific agroecological niches	Superior photosynthate remobilization to pod and grain formation	Superior photosynthate remobilization to pod and grain formation
	Zones with terminal drought stress and soils with lower capacity to store available water deep in the soil profile	Zones with intermittent drought stress and soils that can store greater amount of available water deep in the soil profile

Results from this study indicate that superior grain yield under drought stress in common bean is related with superior root vigor that helps the plant to access water, and to moderate transpiration rates and vegetative growth. Several lines of the water spender type were associated with effective use of water (EUW) probably resulting from a deeper root system, higher canopy biomass production and improved partitioning of photosynthates to grain. A few lines of the water saver type combined higher water use efficiency (WUE) with a relatively shallower root system and better photosynthate partitioning under drought stress. Superior SNF ability under drought stress was related with superior presence of roots with greater values of mean root diameter. Superior N uptake from the soil was associated with a larger root system with more presence of fine roots. Seven lines (SEA 15, NCB 280, SCR 16, SMC 141, BFS 29, BFS 67, SER 119) combined the shoot and root traits of water spending ideotype characterized by superior grain production and a vigorous and deeper root system under drought stress. Four genotypes (RCB 593, SEA 15, NCB 226, BFS 29) that were superior in their SNF ability under drought stress were also identified and these could serve as parents for further improvement of SNF ability and drought resistance of common bean.
