# Supplementary material for: Root traits and their potential links to plant ideotypes to improve drought resistance in common bean
Source: Theor Exp Plant Physiol. 2017 Aug 31;29:143–54. doi: 10.1007/s40626-017-0090-1 (PMC7797623; doi:10.1007/s40626-017-0090-1)
Supplement: Supplementary file 1 [file TEPP-29-143-s001.docx]

Root traits and their potential links to plant ideotypes to improve drought resistance in common bean

Jose Polania, Charlotte Poschenrieder, Idupulapati Rao, Stephen Beebe

Fig. S1 Distributions of 36 bean genotypes under drought stress on the first two principal component axes
